# Molecular functional mechanisms of two alcohol acetyltransferases in *Lavandula x intermedia* (lavandin)

**DOI:** 10.3389/fchem.2025.1627286

**Published:** 2025-06-11

**Authors:** Dafeng Liu, Yanyan Du, Ablikim Abdiriyim, Lvxia Zhang, Daoqi Song, Huashui Deng, Xiongying Wen, Yanyan Zhang, Bingwang Sun

**Affiliations:** ^1^ Xinjiang Key Laboratory of Lavender Conservation and Utilization, College of Biological Sciences and Technology, Yili Normal University, Yining, Xinjiang, China; ^2^ School of Life Sciences, Xiamen University, Xiamen, Fujian, China

**Keywords:** *Lavandula x intermedia* (lavandin), alcohol acetyltransferase, structural prediction, enzyme activity assay, gene expression levels

## Abstract

Volatile esters are key flavor components in most plants, including *Lavandula x intermedia* (lavandin). The final step in ester biosynthesis is catalyzed by *Lavandula x intermedia* alcohol acyltransferases (LiAATases), which attach alcohols to acyl groups. However, the functional role and mechanism of LiAATases remain poorly understood. Here, we predicted their structural models using AlphaFold2 and identified potential active site residues through the GalaxyWEB program. Catalytic assays were optimized at pH 7.5 and 30 °C. Substrate specificity for alcohols was assessed for both enzymes. Gene expression analysis revealed that *LiAATase1* and *LiAATase2* were most highly expressed in the petals and pistils, respectively, with peak expression occurring at stage 4 for *LiAATase1* and stage 1 for *LiAATase2*. Our study aims to elucidate the functional properties of alcohol acyltransferases in *Lavandula x intermedia*, contributing to an understanding of ester biosynthesis and specificity in this species.

## Introduction

Esters are a key class of volatile compounds that play a significant role in the formation of plant aromas (Liu et al., 2023; Aschariyaphotha et al., 2024). Alcohol acyltransferase (AATase) catalyzes the transfer of an acyl group from acyl-CoA to an alcohol, resulting in ester production (Aharoni et al., 2004; Defilippi et al., 2005; Seixas et al., 2023). This enzyme plays a critical role in the biosynthesis of volatile esters in various plant species, including climacteric fruits such as melons, bananas, and apples, as well as non-climacteric plants like strawberries, passionfruit, pineapple, and certain flowers like *Gypsophila*, *Clarkia breweri*, and *Karawek* (Beekwilder et al., 2004; Canessa et al., 2004; Defilippi et al., 2005; Guterman et al., 2006; Khanom and Ueda, 2008; Mascarell-Creus et al., 2009; de Jong et al., 2010; Shah et al., 2012; Ghezzi et al., 2017; Moon et al., 2021; Wang et al., 2021; Liu et al., 2023; Saez et al., 2024).

Plant lavender essential oils (EOs) primarily consist of volatile monoterpenes and sesquiterpenes, which may include alcohols (Patrignani et al., 2021; Wani et al., 2021; Bavarsad et al., 2023; Liu et al., 2024a; Liu et al., 2025c). These oils serve various physiological and ecological functions, such as allelopathy, plant defense, and pollinator attraction (Benabdelkader et al., 2011; Ebadollahi et al., 2020; Karpiński, 2020). Additionally, lavender EOs possess considerable economic importance due to their extensive applications in cosmetics, personal care products and alternative medicine (Lesage-Meessen et al., 2015; de Groot and Schmidt, 2016a; de Groot and Schmidt, 2016b). The enzyme *Lavandula x intermedia* alcohol acetyltransferase (LiAATase) is essential for the biosynthesis of volatile esters by catalyzing the transfer of acyl groups from acyl-CoA to alcohols. LiAATase is part of a larger enzyme family responsible for alcohol acetylation. Despite its importance in aroma production, the function and mechanism of LiAATases in lavender remain poorly understood.

Herein, we used the NPS@ server to predict the secondary structures of LiAATase1 and LiAATase2, and structural models were generated using AlphaFold2. The GalaxyWEB program was employed to identify potential active site residues. Optimal catalytic conditions for both LiAATase1 and LiAATase2 were determined to be pH 7.5 at 30 °C. Alcohol substrate specificities for both enzymes were assessed. Gene expression analysis revealed that *LiAATase1* and *LiAATase2* exhibited the highest expression in the petals and pistils, respectively. Furthermore, peak expression of *LiAATase1* occurred at stage 4, while *LiAATase2* showed highest expression at stage 1, among the five developmental stages analyzed. This study aimed to characterize alcohol acetyltransferases in *Lavandula x intermedia*, contributing to the understanding of their role in ester biosynthesis and specificity.

## Results

### Secondary structure prediction of LiAATase1 and LiAATase2

**FIGURE 1:**
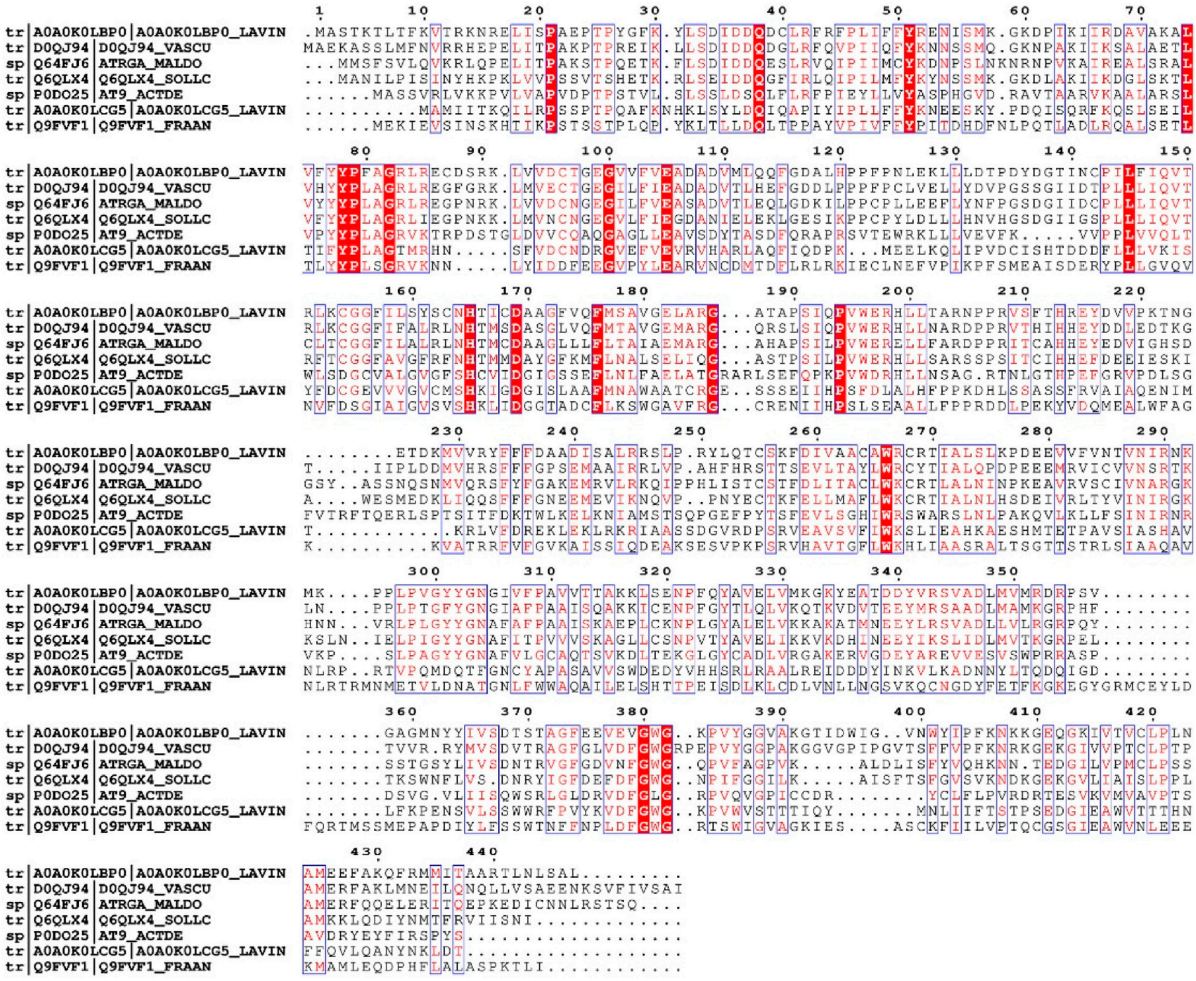
Sequence alignment of alcohol acetyltransferase family. The sequence alignment employs the ClustalW default color scheme, where conserved amino acids are highlighted with more intense colors than non-conserved residues. The reference proteins included in the alignment are as follows: A0A0K0LBP0, *Lavandula x intermedia* (Lavandin, *Lavandula angustifolia* x *Lavandula latifolia*); D0QJ94, *Vasconcellea cundinamarcensis* (Mountain papaya, Carica candamarcensis); Q64FJ6, *Malus domestica* (Apple, Pyrus malus); Q6QLX4, *Solanum lycopersicum* (Tomato, Lycopersicon esculentum); P0DO25, *Actinidia deliciosa* (Kiwi); A0A0K0LCG5, *Lavandula x intermedia* (Lavandin, *Lavandula angustifolia* x *Lavandula latifolia*); Q9FVF1, *Fragaria ananassa* (Strawberry, *Fragaria chiloensis* x *Fragaria virginiana*).

**FIGURE 2:**
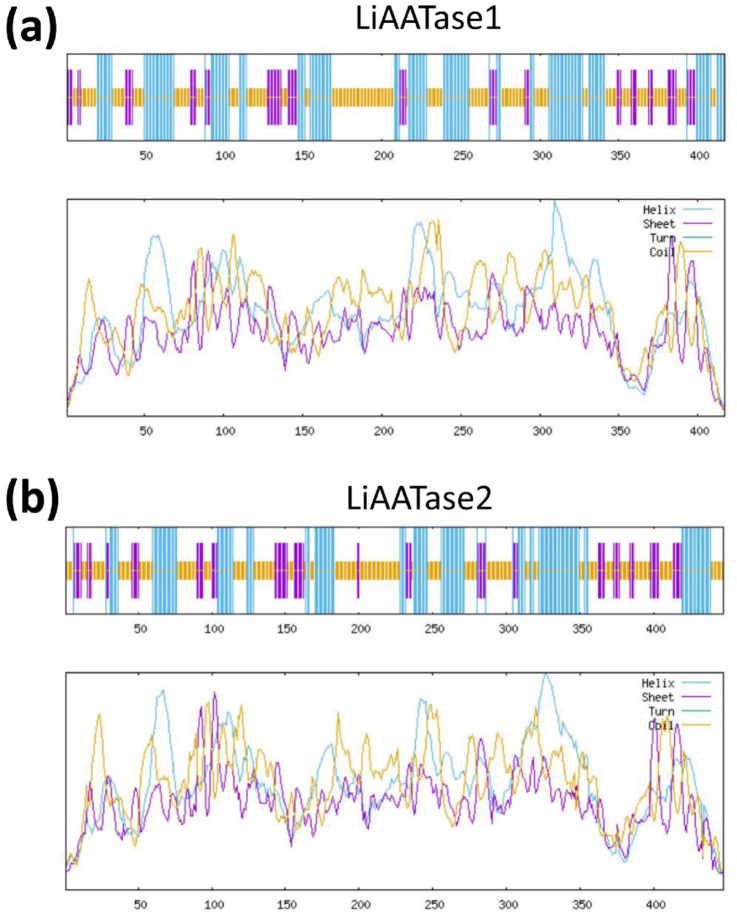
Prediction of secondary structure models of LiAATases (*Lavandula x intermedia* alcohol acyltransferases). The secondary structure predictions for LiAATase1 and LiAATase2 are shown in **(a,b)** respectively.

Using the amino acid sequences of LiAATase1 (UniProt code A0A0K0LCG5) and LiAATase2 (UniProt code A0A0K0LBP0) (Figure 1), we predicted their secondary structures *via* the NPS@ server (Combet et al., 2000). The predicted secondary structures of LiAATase1 and LiAATase2 consist of alpha helices (37.17% and 33.78% of residues, respectively), along with multiple strands and coils (Figure 2). LiAATase1 contains 155 residues in helices, while LiAATase2 has 151.

### Prediction and quality assessment of LiAATase1 and LiAATase2 structures

The three-dimensional (3D) structures of LiAATase1 and LiAATase2 were predicted using AlphaFold2 (Jumper et al., 2021; Wayment-Steele et al., 2023), a deep learning-based tool that provides highly accurate and reliable protein structure predictions, surpassing traditional homology modeling methods.

**FIGURE 3:**
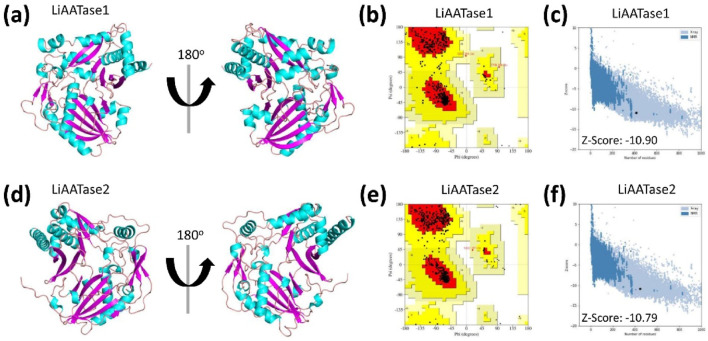
Structural prediction and quality assessment of LiAATase1 and LiAATase2. The three-dimensional (3D) structures of **(a)** LiAATase1 and **(d)** LiAATase2 were predicted using AlphaFold2. Both models are depicted as cyan ribbon diagrams from two distinct orientations, with α-helices in pink and β-sheets in cyan. Structural validation was performed using Ramachandran plot analysis [**(b)** for LiAATase1, **(e)** for LiAATase2], where the most favorable residue conformations are highlighted in red, and less favorable regions are shown in progressively lighter shades. Additionally, **(c, f)** ProSA analysis yielded Z-scores of −10.90 (LiAATase1) and −5.68 (LiAATase2), confirming the high quality of the predicted models.

To evaluate the quality of the predicted structures (Figures 3a,d), we employed the Ramachandran plot to analyze the dihedral angles of the protein backbones, ensuring they fell within acceptable regions, which indicates a valid protein conformation. For LiAATase1, 88.7% of the residues were in the most favored region, 10.8% in the additionally allowed region, 0.5% in the generously allowed region, and none in the disallowed region (Figure 3b; Table 1). For LiAATase2, 90.5% of residues were in the most favored region, 9.2% in the additionally allowed region, 0.3% in the generously allowed region, and none in the disallowed region (Figure 3e; Table 1).

**TABLE 1:** Ramchandran plot analysis of structural models of the two alcohol acetyltransferases using PDBsum.

Constructs	Residues in most favored regions		Residues in additional allowed regions		Residues in generously allowed regions		Residues in disallowed regions	
Residues	Number of residues	% of residues	Number of residues	% of residues	Number of residues	% of residues	Number of residues	% of residues
LiAATase1^a^	338	88.7	41	10.8	2	0.5	0	0
LiAATase2^b^	353	90.5	36	9.2	1	0.3	0	0

^a^ Number of end-residues (excl. Gly and Pro): 2; Number of glycine residues (shown as triangles): 13; Number of proline residues: 21.

^b^ Number of end-residues (excl. Gly and Pro): 2; Number of glycine residues (shown as triangles): 28; Number of proline residues: 27.

ProSA analysis of the models showed Z-scores of −10.90 for LiAATase1 and -10.79 for LiAATase2 (Figures 3c,f). Both Z-scores were well below −10.00, demonstrating excellent model quality. However, the slight difference between the two Z-scores does not suggest a significant structural discrepancy in the model.

**FIGURE 4:**
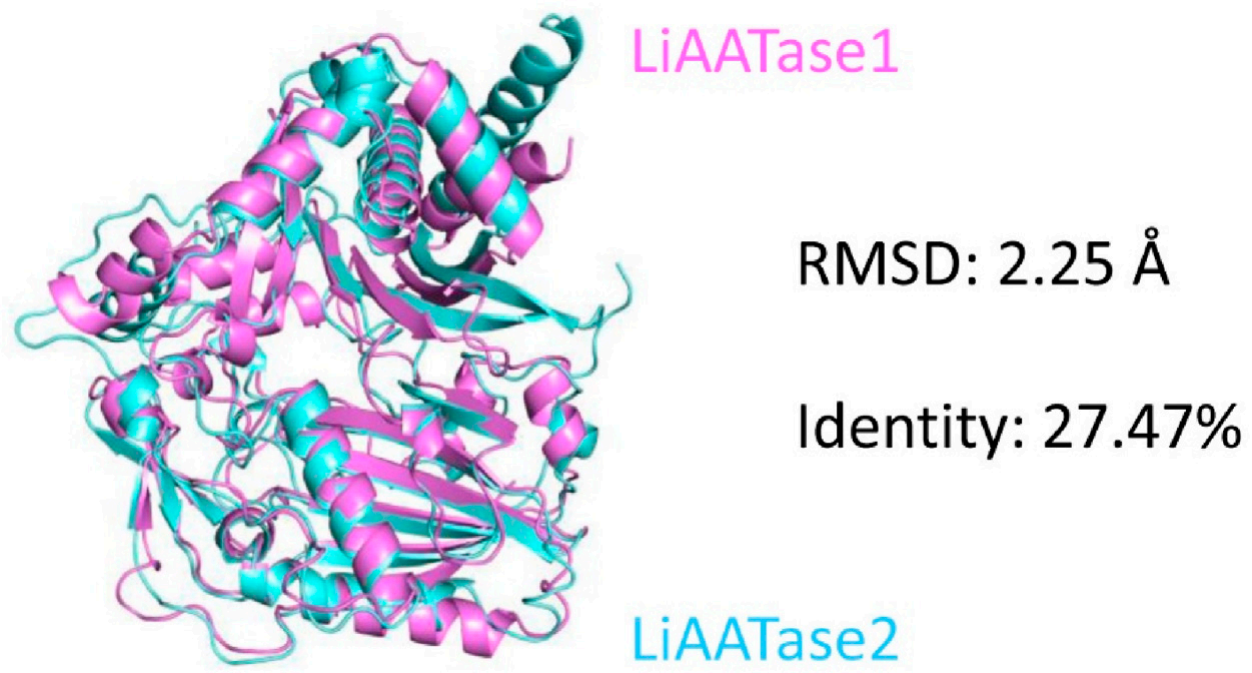
Comparative structural analysis of LiAATase1 (in magenta) and LiAATase2 (in cyan). LiAATase1 adopted an overall fold similar to that of LiAATase2, but the root mean square deviation (RMSD) value for all atoms was large (2.25 Å), and the amino acid sequence identity was only 27.47%.

Although the overall fold of LiAATase1 closely resembles that of LiAATase2 (Figure 4), the root mean square deviation (RMSD) for all atoms was 2.25 Å, and the sequence identity was 27.47% (Figure 4).

### Predicting LiAATase1 and LiAATase2 active sites

**FIGURE 5:**
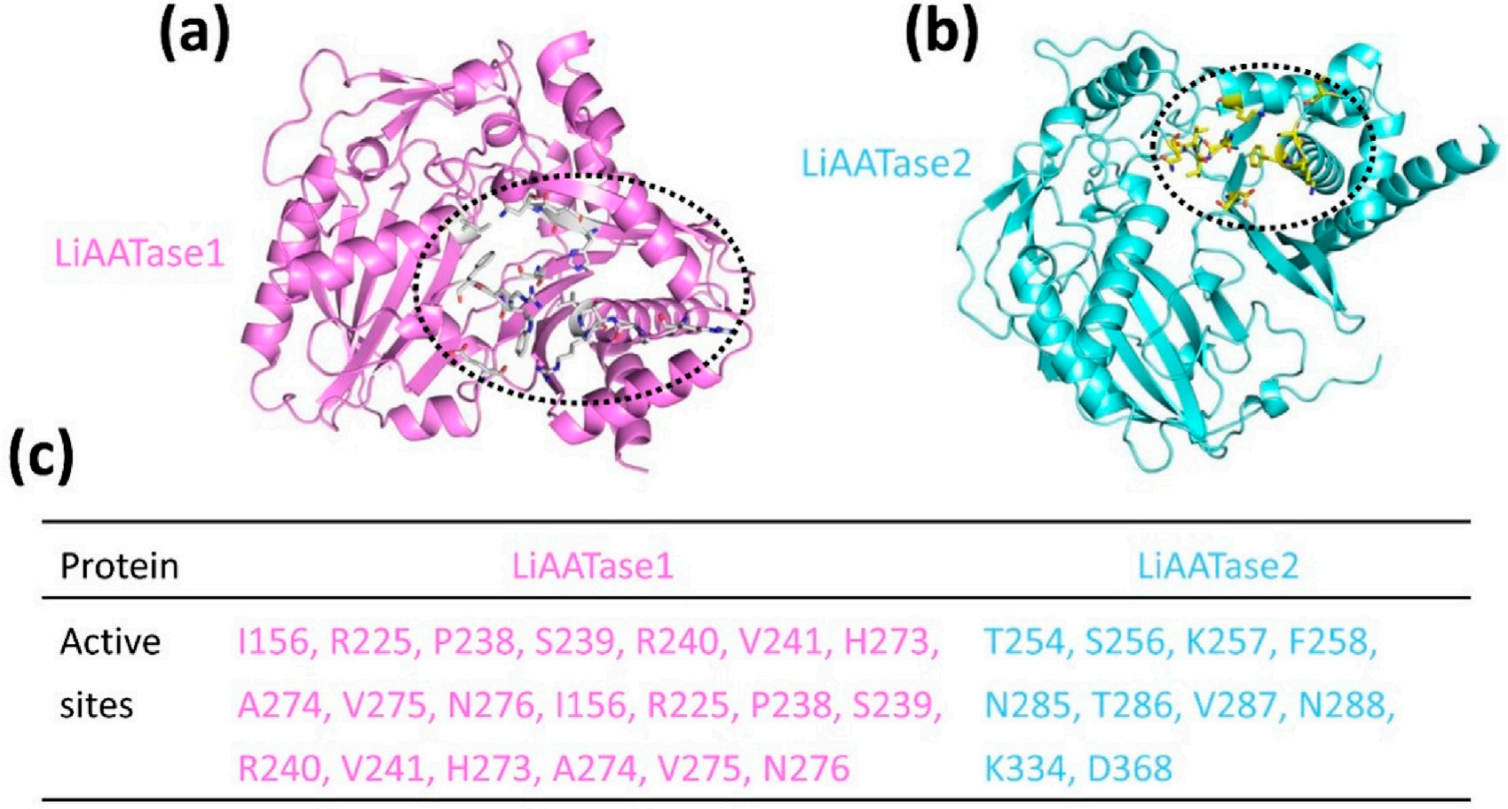
Predicting **(a)** LiAATase1 and **(b)** LiAATase2 active site residues using the GalaxyWEB program. LiAATase1 and LiAATase2 were colored in magenta and cyan, respectively. **(c)** The residues in the active site of LiAATase1 and LiAATase2.

Using the predicted models (Figures 3a,d, 4, 5a,b), we employed the GalaxyWEB program (Ko et al., 2012; Heo et al., 2013; Heo et al., 2016; Seok et al., 2021) to identify the active sites of LiAATase1 and LiAATase2. The analysis revealed that the active site residues of LiAATase1 include I156, R225, P238, S239, R240, V241, H273, A274, V275, N276 (Figures 5a,c). For LiAATase2, the active site residues are T254, S256, K257, F258, N285, T286, V287, N288, K334, and D368 (Figures 5b,c). These residues are likely involved in interactions with the alcohol substrate, potentially forming bonds with the substrate’s side chain atoms.

### Effects of temperature and pH on the activity of LiAATase1 and LiAATase2

**FIGURE 6:**
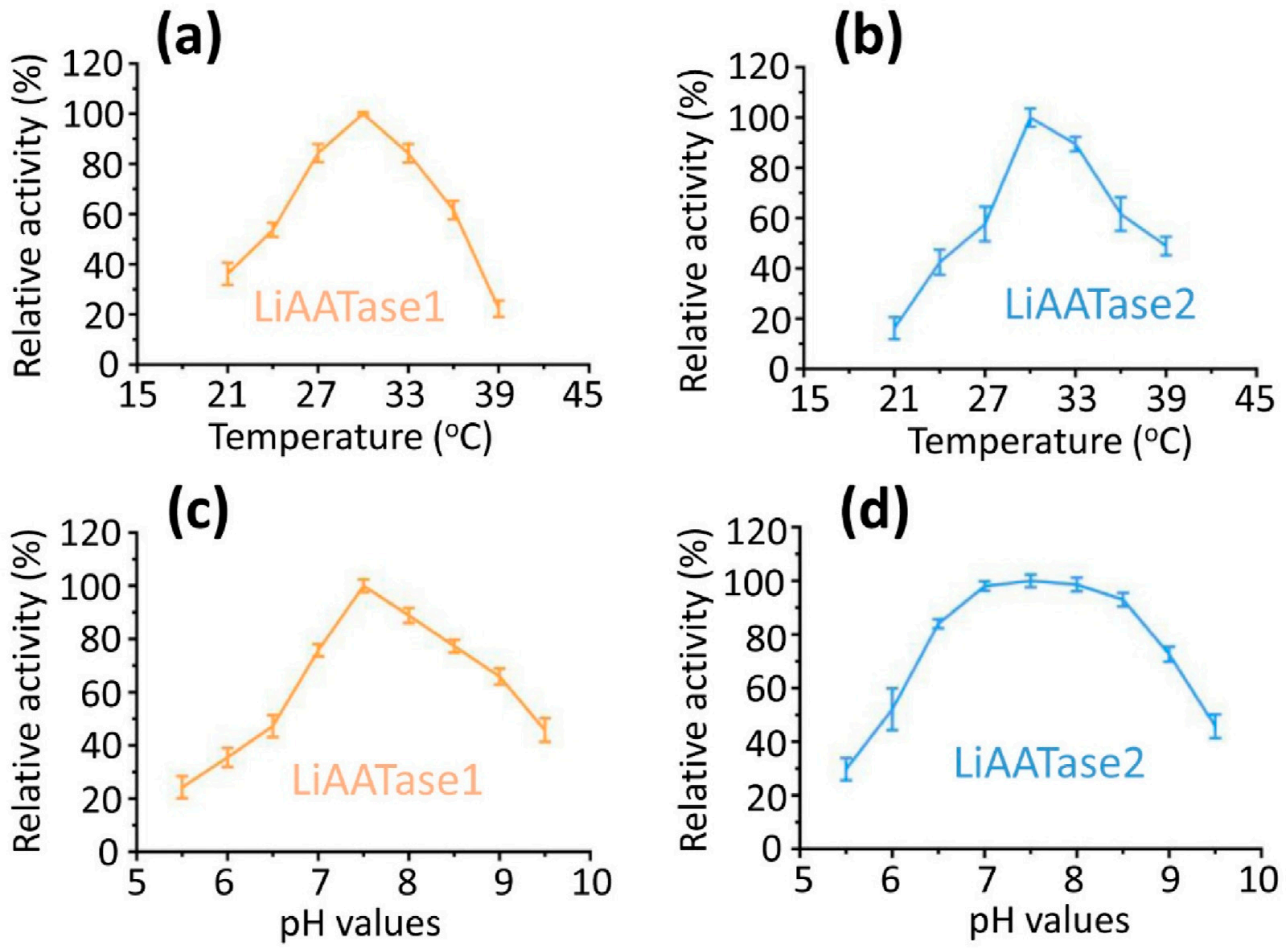
The effects of temperature and pH on the relative activity (%) of LiATTase1 and LiATTase2. Both enzymes exhibited maximal activity at 30 °C **(a,b)**. Similarly, their optimal pH was 7.5 **(c,d)**.

To determine the optimal conditions for alcohol acetyltransferase activity of LiAATase1 and LiAATase2, we examined the effects of temperature and pH on enzyme activity (Figure 6). Activity was tested across a temperature range of 21–39°C, revealing an increase in activity up to 30°C, after which it declined (Figures 6a,b). The enzymes showed optimal activity within a pH range of 5.5–9.5, with peak activity occurring at pH 7.5 before decreasing (Figures 6c,d). Based on these results, we selected pH 7.5 and 30 °C as the optimal conditions for further activity assessments.

### Alcohol substrate specificities of LiAATase1 and LiAATase2

**FIGURE 7:**
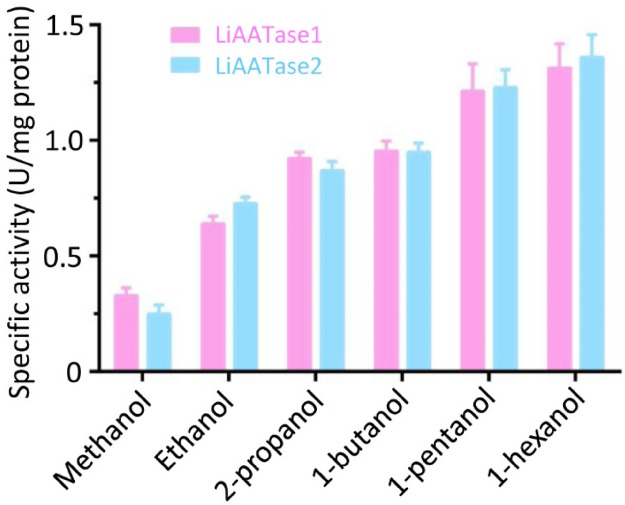
Specific activity and relative activity of LiAATase1 and LiATTase2 using different alcohols. The catalytic activity of both enzymes was determined under pH 7.5 and 30 °C.

Alcohol acetyltransferases are well-known for their role in producing acetate esters and are often associated with ethylene-dependent or ripening-specific processes in many plants (Beekwilder et al., 2004; Defilippi et al., 2005; Aschariyaphotha et al., 2024). The activity of LiAATases was determined under the optimal conditions (pH 7.5 at 30 °C). We found that both LiAATase1 and LiAATase2 demonstrated high activity with medium-chain alcohols, particularly 1-hexanol, while showing minimal activity with short-chain alcohols like methanol and ethanol. The enzyme activity increased with the length of the alcohol chain, with 1-hexanol exhibiting 4.1 to 5.5 times higher activity than methanol (Figure 7). This trend may be attributed to alcohol acyltransferase’s preference for utilizing acyl-CoA residues, as the enzyme tends to react more readily with acyl-CoA substitutes other than acetyl-CoA.

### Spatial and temporal analyses of genes LiAATase1 and LiAATase2

**FIGURE 8:**
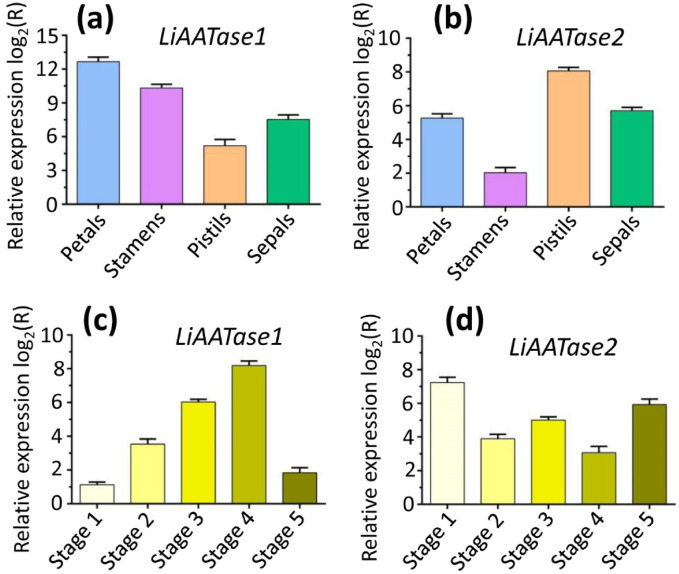
Gene expression profiles of *LiAATase1* and *LiAATase2*. **(a,b)** Expression levels of genes *LiAATase1* and *LiAATase2* in different flower organs (petals, stamens, pistils and sepals). **(c,d)** Expression levels of *LiAATase1* and *LiAATase2* genes during different flowering stages. Flower development was classified into five stages, i.e., flowers with tightly closed buds (stage 1), flowers with slightly open buds (stage 2), flowers beginning to open (stage 3), flowers in full bloom (stage 4), and flowers completely wilted (stage 5). The relative expression levels of genes *LiAATase1* and *LiAATase2* were quantified using RT-qPCR (real-time quantitative polymerase chain reaction). Expression ratios are shown as log_2_ values, and values above zero indicate upregulation of gene expression.

To establish a detailed spatial and temporal expression profile, RT-qPCR (real-time quantitative polymerase chain reaction) was used to quantify the expression levels of the target genes LiAATase1 and LiAATase2 across different flower organs and developmental stages. The results showed that LiAATase1 expression was highest in the petals (Figure 8a), while LiAATase2 expression peaked in the pistils (Figure 8b). Regarding developmental stages, LiAATase1 expression was greatest at stage 4 (Figure 8c), whereas LiAATase2 expression was highest at stage 1 (Figure 8d). This tissue- and stage-specific expression pattern highlights the significant role of LiAATase1 and LiAATase2 in lavender essential oil biosynthesis, with flower tissues being the primary site of expression.

## Discussion

In this work, the secondary structures of LiAATase1 and LiAATase2 were predicted using the NPS@ server, and their structural models were generated with AlphaFold2. Active site residues were identified through the GalaxyWEB program. Optimal catalytic conditions for both enzymes were determined to be pH 7.5 and 30 °C. Substrate specificity assays revealed the alcohol preferences of LiAATase1 and LiAATase2. Gene expression analysis indicated that *LiAATase1* was most highly expressed in petals, while *LiAATase2* showed peak expression in pistils. Additionally, *LiAATase1* had its highest expression at stage 4, whereas *LiAATase2* peaked at stage 1 among the five developmental stages examined. This study highlights key characteristics of alcohol acetyltransferases in lavender, offering insights into their roles in ester biosynthesis and specificity in *Lavandula x intermedia*.

Alcohol acetyltransferase (EC 2.3.1.84) plays a crucial role in aroma biosynthesis by catalyzing the formation of esters from acyl-CoA and alcohols (Beekwilder et al., 2004; Defilippi et al., 2005; Khanom and Ueda, 2008). Despite their low sequence identity, alcohol acetyltransferases share highly conserved three-dimensional structures, with partially conserved active sites. These active sites bind acyl-CoA and alcohol, generally featuring an HXXXXD motif that forms a substrate reaction channel (Bayer et al., 2004; Galaz et al., 2013; Zheng et al., 2016). These differ from LiAATases, suggesting that alcohol acetyltransferases may employ distinct catalytic mechanisms. To further investigate these, we are exploring the structural and mechanistic features of LiAATase-catalyzed reactions using experimental approaches such as crystallography. This structural arrangement allows independent binding of the substrate and co-substrate, catalyzing C-O bond formation and promoting the synthesis of corresponding ester compounds (Bayer et al., 2004; Ma et al., 2005; Morales-Quintana et al., 2011; Cumplido-Laso et al., 2012; Morales-Quintana et al., 2012; Galaz et al., 2013; Morales-Quintana et al., 2013; Morales-Quintana et al., 2015; Zheng et al., 2016; Liu et al., 2023; Saez et al., 2024). These enzymes are capable of pairing various alcohols with acyl-CoA, resulting in the production of a diverse array of esters, which contributes to the complexity of ester profiles (Horton et al., 2003; Guterman et al., 2006; Lilly et al., 2006). The alcohol component of an ester corresponds to the alcohols primarily synthesized in the plant, while the acyl component reflects the specificity of the alcohol acetyltransferases for different acyl-CoA molecules. For instance, strawberry alcohol acetyltransferase shows strong activity with hexanol and both acetyl- and butyl-CoA, while banana alcohol acetyltransferase is highly active with butanol and acetyl-CoA but less so with butyl-CoA (Zhang et al., 2014; Hu et al., 2016). Therefore, alcohol acetyltransferases from different plants exhibit unique substrate specificities for both alcohols and acyl-CoA.

Additionally, alcohol acetyltransferase has also been identified in the native Californian flower *C. breweri*, where it plays a key role in the esterification of benzyl alcohols within the flower (Defilippi et al., 2005; Wang et al., 2021; Liu et al., 2023). A strong affinity for aromatic alcohols, including benzyl alcohol and cinnamyl alcohol. Benzyl acetate, a significant compound in the fragrance industry, is a prominent aroma in this flower. It is the primary component of jasmine and gardenia essential oils and is commonly used as a minor constituent in various other oils.

The engineering of volatile emissions in plants has primarily focused on terpenoids. For example, a tomato variety with low endogenous linalool levels in the fruit was transformed with *C. breweri* linalool synthase under the control of the fruit-specific E8 promoter (Beekwilder et al., 2004). This led to significantly elevated levels of S-linalool in the fruit. The same gene was introduced into carnation flowers to induce linalool emission. However, overexpression of *C. breweri* linalool synthase in carnations resulted in the accumulation of linalool as a nonvolatile glucopyranoside conjugate, suggesting that endogenous enzymes in petunia sequester volatile linalool as a nonvolatile form (Moon et al., 2021; Lee and Trinh, 2022). In Arabidopsis leaves constitutively expressing a strawberry linalool synthase, linalool production occurred alongside its glycosylated and hydroxylated derivatives. Furthermore, overexpression of alcohol dehydrogenase in tomato fruit increased the levels of hexanol and cis-3-hexenol, at the expense of aldehyde production (Beekwilder et al., 2004).

In conclusion, our study presents a novel approach to comprehensively explore the functional mechanisms of alcohol acetyltransferases in *Lavandula x intermedia*, aiming to improve the quality of lavender essential oils.

## Materials and methods

### Bioinformatics analysis

The amino acid sequences of LiAATase1 (UniProt code A0A0K0LCG5) and LiAATase2 (UniProt code A0A0K0LBP0) were analyzed using the ProtParam online server (https://web.expasy.org/protparam/) to predict their chemical properties and physicochemical parameters.

### Prediction of structural models

Structural predictions for LiAATase1 and LiAATase2 were performed using the AlphaFold2 program (Jumper et al., 2021; Wayment-Steele et al., 2023). Secondary structures were predicted with the NPS@ server (Combet et al., 2000), while active site residues were identified using the GalaxyWEB program (Ko et al., 2012; Heo et al., 2013; Heo et al., 2016; Seok et al., 2021). Multiple sequence alignment data were obtained from the LSQKAB program within the CCP4 suite (Collaborative Computational Project, 1994), and the root mean square deviation (RMSD) for Cα atoms was calculated. Structural images were generated using PyMOL 2.3.4 (https://www.pymol.org/2/).

### Quality assessment of structural models

To validate the tertiary structures, Ramachandran plots for LiAATase1 and LiAATase2 were generated using the PDBsum database (Laskowski, 2004; de Beer et al., 2014; Laskowski et al., 2017; Laskowski, 2022). This tool assesses protein structure quality by detecting geometric errors, thereby improving the accuracy of the models. The Ramachandran plot specifically evaluates the stereochemical properties of the structures by displaying the dihedral angles of amino acid residues, highlighting the allowed conformational regions and identifying disallowed orientations.

Additionally, ProSA (Protein Structure Analysis) is a widely used tool for analyzing and validating predicted protein models (Wiederstein and Sippl, 2007). The z-score reflects overall model quality and is plotted against the z-scores of all experimentally determined protein chains in the current PDB. The plot distinguishes between structure groups (e.g., X-ray, NMR) using color-coding. This allows assessment of whether the input structure’s z-score falls within the expected range for native proteins of comparable size.

### Protein isolation and purification

LiAATase1 and LiAATase2 were isolated and purified with slight modifications to previously described methods (Egea-Cortines et al., 2019; Nazeer et al., 2019). Fresh flowers were first washed with tap water, then with distilled water, and dried in the shade for 5–6 days at room temperature. The dried samples were powdered in liquid nitrogen using a mortar and pestle. Total protein was extracted using a Tris-buffer saline solution (150 mM NaCl, 20 mM Tris-HCl, PVP, pH 7.4), with a slight adjustment to the extraction buffer ratio (1:7, w/v). The sample was filtered through three layers of muslin cloth, and the filtrate was stirred magnetically at 4 °C overnight. The sample was then centrifuged at 18,000 rpm for 30 min at 4°C, and the supernatant was collected while discarding the pellet.

For protein purification, the crude extract was precipitated using varying ammonium sulfate saturation levels (20%, 35%, 55%, 75%, and 90%). A 75% saturated ammonium sulfate solution yielded the best quality pellet after centrifugation at 18,000 rpm for 30 min at 4 °C. The supernatant was then precipitated with 75% ammonium sulfate saturation and stored at −20 °C overnight to ensure complete protein precipitation. The following morning, the sample was centrifuged again at 18,000 rpm for 30 min at 4°C, with the supernatant discarded. The resulting pellet was washed six times with acetone and then dried. The protein was dialyzed against distilled water for 12 h at 4°C, and purified using size exclusion chromatography (SEC).

### Enzymatic activity assays

Enzyme assays were conducted in 25-mL glass syringes with Luer lock caps, following a previously described method with minor modifications (Hu et al., 2016; Stribny et al., 2016; Egea-Cortines et al., 2019). The reaction mixtures contained a glycerol buffer (50 mM potassium phosphate, pH 7.5, 10% (w/v) glycerol), which included higher alcohol as a co-substrate, acetyl-CoA as the second co-substrate, and a cell extract. The volume ratio of higher alcohol, acetyl-CoA, and cell extract was 10:1:4, with a final reaction volume of 1.5 mL. Isoamyl alcohol (0.01–100 mM final concentration), isobutanol (60 mM), or 2-phenylethanol (30 mM) were used as substrates, along with 0.8 mM acetyl-CoA. Substrate concentrations were carefully measured. After adding all components, entrapped air was removed using the plunger, and the syringe was attached to an orbital shaker. Following a 30-min incubation, 1.5-mL samples were transferred to 15-mL vials containing 0.35 g NaCl for ester quantification *via* gas chromatography. Enzyme activity was terminated by adding 60 mL of a saturated KSCN solution.

### RT-qPCR analysis of gene expression levels

Gene expression of *LiAATase1* and *LiAATase2* was quantified using real-time quantitative polymerase chain reaction (RT-qPCR) with PowerUp SYBR Green Master Mix (Applied Biosystems). Total RNA was extracted from various developmental stages and tissues using the Universal Plant Total RNA Extraction Kit (Bioteke, Beijing, China) according to the manufacturer’s instructions. cDNA was synthesized from the RNA samples using the PrimeScript first Strand cDNA Synthesis Kit (Takara, Kyoto, Japan). The primers used in the experiments are listed in Table 2. RT-qPCR analysis was performed on an Applied Biosystems QuantStudio five instrument. Data were analyzed using the 2^−ΔΔCT^ method (Livak and Schmittgen, 2001; Schmittgen and Livak, 2008; Hawkins and Guest, 2017; Green and Sambrook, 2018; Liu et al., 2024a; Liu et al., 2024b; Liu et al., 2025a; Liu et al., 2025b; Liu et al., 2025c), and relative expression ratios were presented as log_2_ values in histograms. A ratio greater than zero indicated upregulation, while a ratio less than zero indicated downregulation. Beta-actin was used as a reference gene for normalization, and a positive control with beta-actin was included in the analysis.

**TABLE 2:** Primers used for RT-qPCR in this study.

Constructs	Primers	Primer sequence (5′-3′)
*Beta-actin*	Forward primer	ggc​agt​ttg​gac​aag​aga​aga​cac​agt​cac​c
	Reverse primer	ttt​ttt​gat​taa​aaa​aaa​aag​ctg​aaa​tca​taa​tat​ttt​taa​t
*LiAATase1*	Forward primer	atg​gcg​atg​att​att​aca​aaa​caa​att​ttg​agg​cca​tca​tct​c
	Reverse primer	tca​agt​atc​caa​ttt​att​gta​att​ggc​ttg​gag​cac​ttg​gaa​g
*LiAATase2*	Forward primer	atg​gca​tcc​acc​aaa​acc​ctg​acc​ttc
	Reverse primer	tca​caa​tgc​tga​aag​att​gag​agt​cct​ggc​ag

### Statistical analysis

All experiments were conducted at least in triplicate. The data were expressed as mean ± SD. Statistical analysis was conducted using Origin 8.5, Microsoft Excel 2013 and SPSS 19.0. In the all statistical evaluations, *p* < 0.05 was considered statistically significant, and *p* < 0.01 was considered high statistically significant.

## Data Availability

The datasets generated in this study are available in online repositories. Details regarding the repository names and accession numbers are provided in the article and Supplementary Material.
