# Supplementary material for: Molecular functional mechanisms of two alcohol acetyltransferases in Lavandula x intermedia (lavandin)
Source: Front Chem. 2025 Jun 11;13:1627286. doi: 10.3389/fchem.2025.1627286 (PMC12187743; doi:10.3389/fchem.2025.1627286)
Supplement: Supplementary file 1 [file DataSheet1.pdf]

ProSA-web
Protein Structure Analysis

Please upload a structure in PDB format: HELP

选择文件 未选择任何文件

Alternatively you can specify a structure by entering its PDB code, chain identifier and NMR model number:

PDB CODE:

PDB CHAIN ID:

PDB MODEL NUMBER:

If you leave the fields for chain id or model number blank,
the first chain of the first model found in the PDB file will be analysed.

Analyse

Results for LiAAT-1.pdb, chain A, model 1 (417 aa)

Overall model quality

HELP

Z-Score: **-10.9**

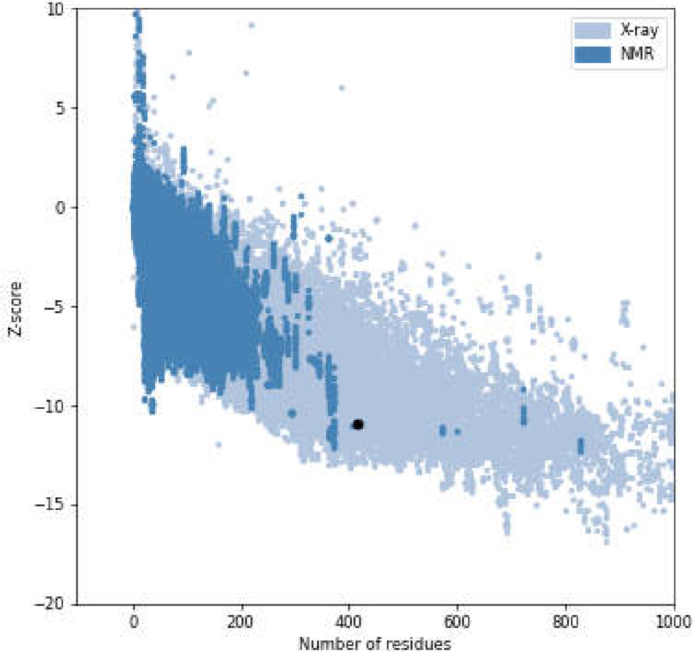

Local model quality

HELP
PNG

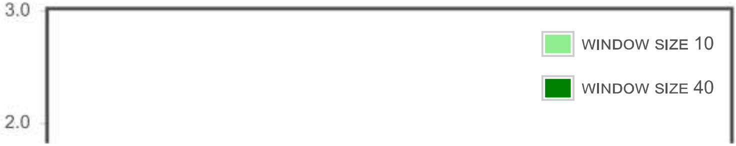

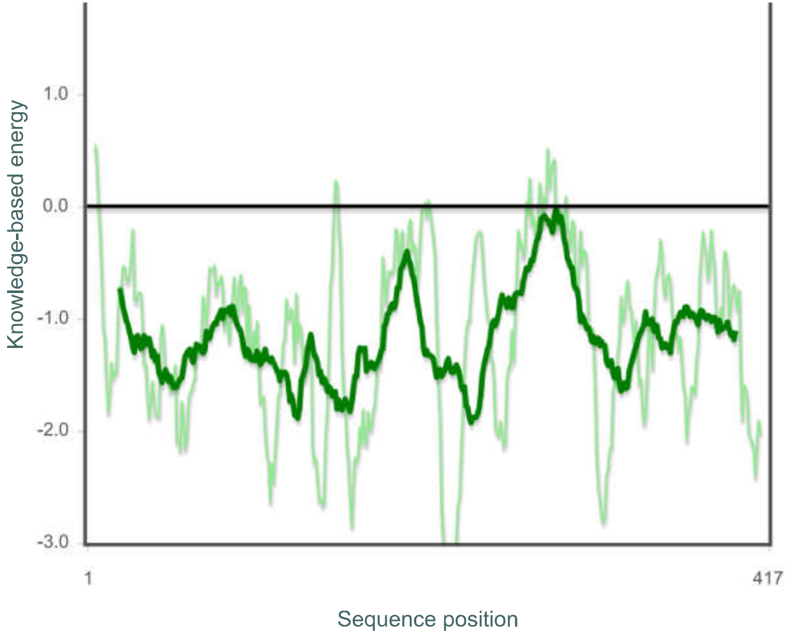
Knowledge-based energy
1.0
0.0
-1.0
-2.0
-3.0
1
417
Sequence position

Pl

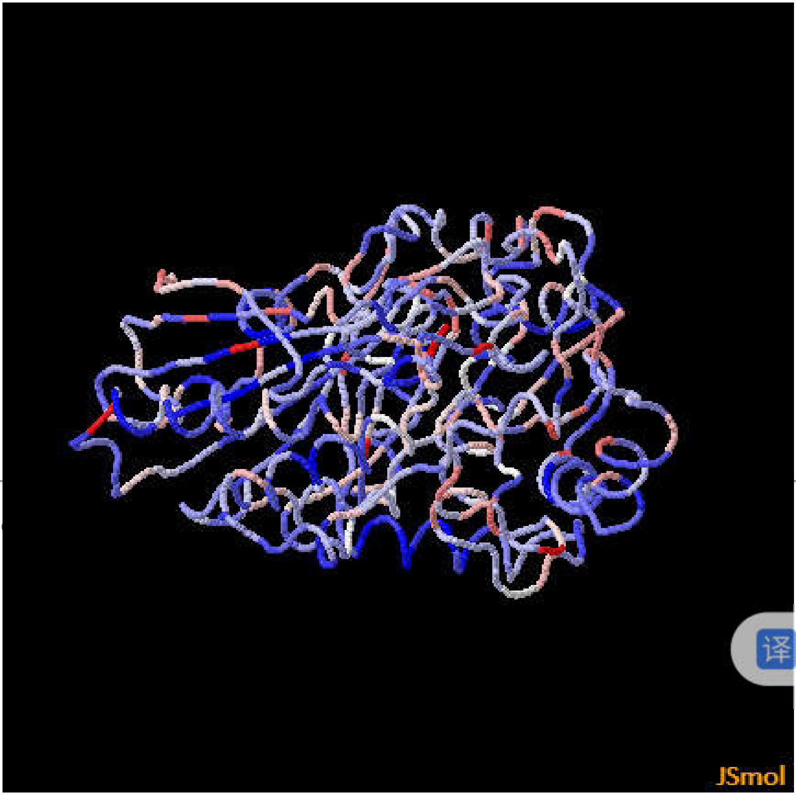

This site is maintained by Markus Wiederstein. For comments and suggestions please contact prosa@came.sbg.ac.at.

UCLA-DOE LAB — SAVES v6.1

← Control panel ↑ New Job

job #153861: LiAAT-1.pdb

ERRAT VERIFY3D WHATCHECK PROCHECK

PROCHECK

Out of 9 evaluations

- Errors: 0
- Warning: 7
- Pass: 2

The evaluations are the '+' (Warning) and '' (Error) in the summary. The categories on the left do not always correspond in number due to PROCHECK output documents.*

Summary	
Ramachandran plot	**Warning**
All Ramachandrans	**Warning**
Chi1-chi2 plots	**Pass**
Main-chain params	
Side-chain params	**Warning**
Residue properties	**Warning**
Bond len/angle	**Warning**
M/c bond lengths	
M/c bond angles	
Planar groups	**Warning**
Program output	

Main Ramachandran plot

- Page
- PDF
- PostScript

PROCHECK

Ramachandran Plot

saves

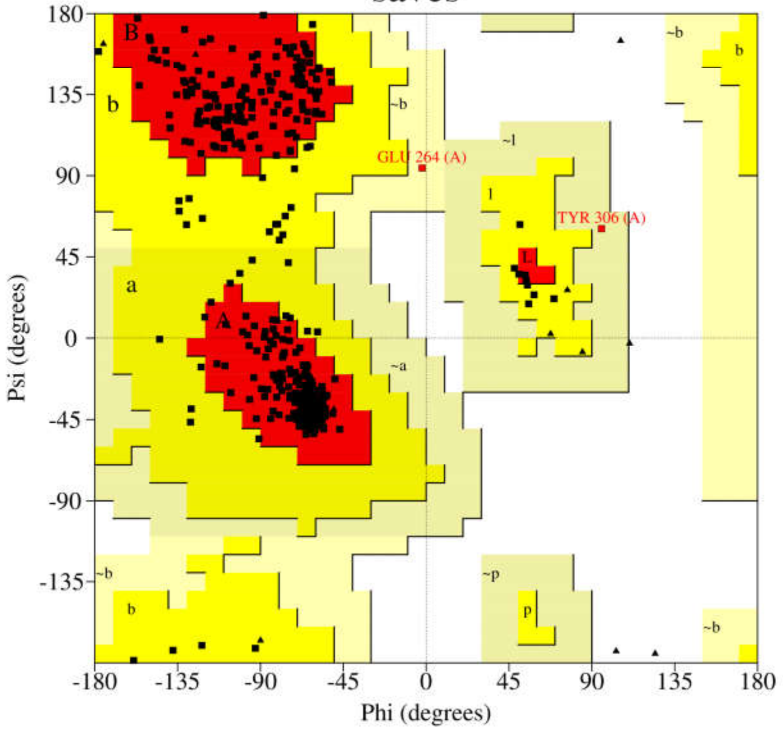

Plot statistics

Residues in most favoured regions [A,B,L]	338	88.7%
Residues in additional allowed regions [a,b,l,p]	41	10.8%
Residues in generously allowed regions [~a,~b,~l,~p]	2	0.5%
Residues in disallowed regions	0	0.0%
	----	------
Number of non-glycine and non-proline residues	381	100.0%
Number of end-residues (excl. Gly and Pro)	2	
Number of glycine residues (shown as triangles)	13	
Number of proline residues	21	

Total number of residues	417	

Based on an analysis of 118 structures of resolution of at least 2.0 Angstroms and R-factor no greater than 20%, a good quality model would be expected to have over 90% in the most favoured regions.

saves_01.ps

Complete. Time taken: 00:07:19

1. *PROVE has not been run*
2. *CRYST has not been run*

saves @2025 - DOE-MBI Services • Usage
Contact: holton at mbi.ucla.edu for any questions

ProSA-web
Protein Structure Analysis

Please upload a structure in PDB format: HELP

选择文件 未选择任何文件

Alternatively you can specify a structure by entering its PDB code,
chain identifier and NMR model number:

PDB CODE:

PDB CHAIN ID:

PDB MODEL NUMBER:

If you leave the fields for chain id or model number blank,
the first chain of the first model found in the PDB file will be analysed.

Analyse

Results for LiAAT-2.pdb, chain A, model 1 (447 aa)

Overall model quality HELP

Z-Score: **-10.79**

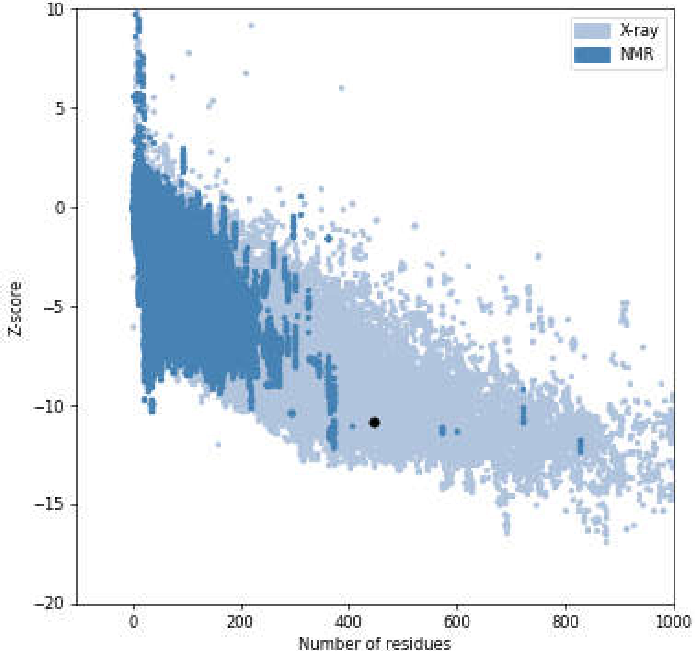

Local model quality

HELP
PNG

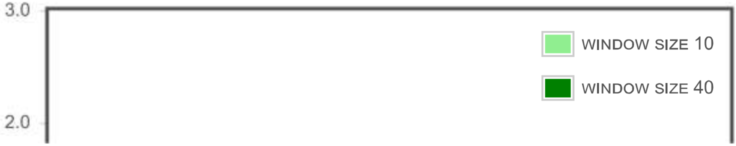

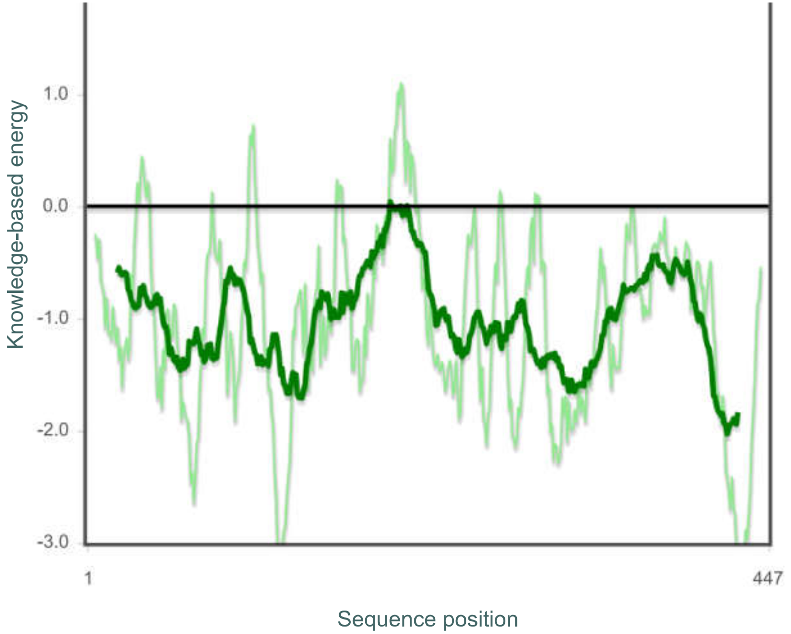
1.0
0.0
-1.0
-2.0
-3.0
Knowledge-based energy
1
447
Sequence position

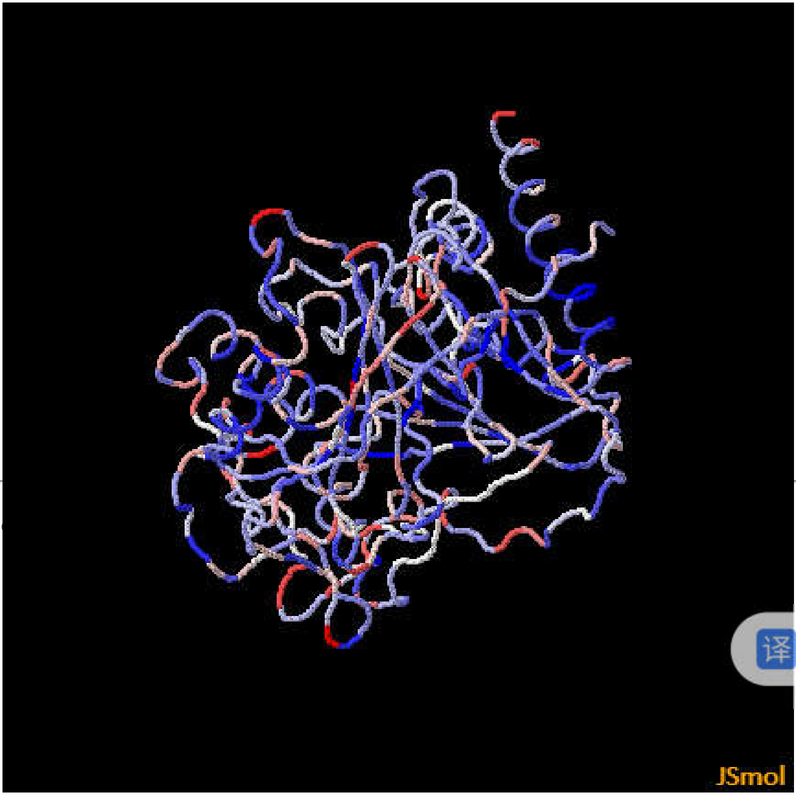

Pl

This site is maintained by Markus Wiederstein. For comments and suggestions please contact prosa@came.sbg.ac.at.

UCLA-DOE LAB — SAVES v6.1

← Control panel ↑ New Job

job #153862: LiAAT-2.pdb

ERRAT VERIFY3D WHATCHECK PROCHECK

PROCHECK

Out of 9 evaluations

- Errors: 0
- Warning: 7
- Pass: 2

The evaluations are the '+' (Warning) and '' (Error) in the summary. The categories on the left do not always correspond in number due to PROCHECK output documents.*

Summary	
Ramachandran plot	**Warning**
All Ramachandrans	**Warning**
Chi1-chi2 plots	**Pass**
Main-chain params	
Side-chain params	**Warning**
Residue properties	**Warning**
Bond len/angle	**Warning**
M/c bond lengths	
M/c bond angles	
Planar groups	**Warning**
Program output	

Main Ramachandran plot

- Page
- PDF
- PostScript

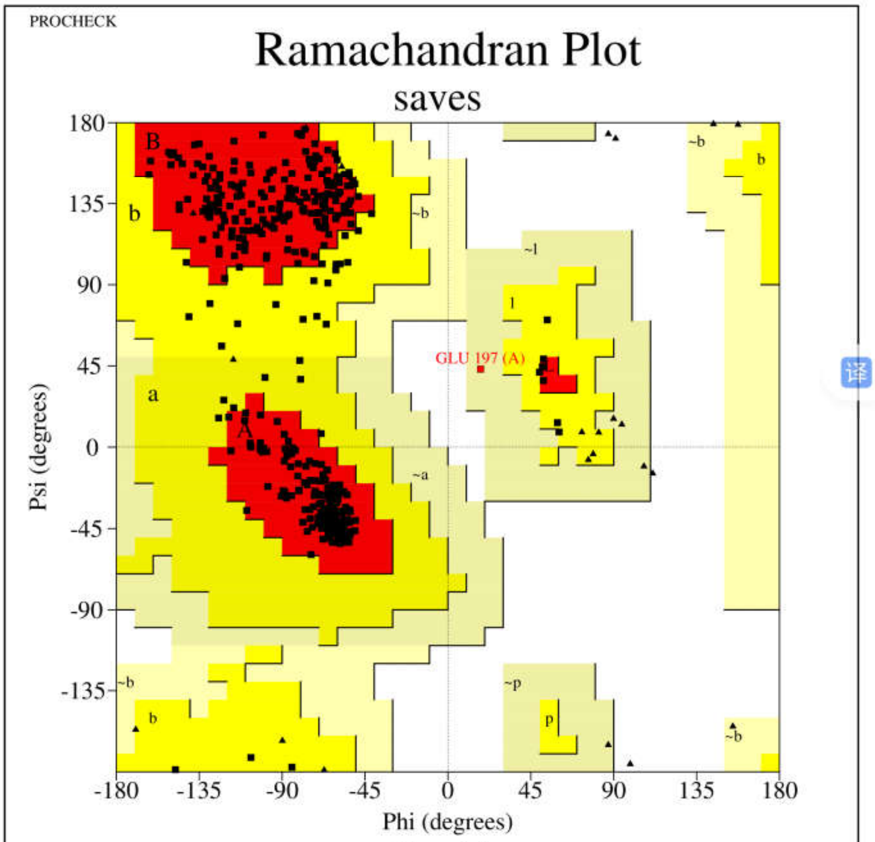

Plot statistics

Residues in most favoured regions [A,B,L]	353	90.5%
Residues in additional allowed regions [a,b,l,p]	36	9.2%
Residues in generously allowed regions [~a,~b,~l,~p]	1	0.3%
Residues in disallowed regions	0	0.0%
	----	------
Number of non-glycine and non-proline residues	390	100.0%
Number of end-residues (excl. Gly and Pro)	2	
Number of glycine residues (shown as triangles)	28	
Number of proline residues	27	

Total number of residues	447	

Based on an analysis of 118 structures of resolution of at least 2.0 Angstroms and R-factor no greater than 20%, a good quality model would be expected to have over 90% in the most favoured regions.

saves_01.ps

Complete. Time taken: 00:06:29

1. *PROVE has not been run*
2. *CRYST has not been run*

saves @2025 - DOE-MBI Services • Usage
Contact: holton at mbi.ucla.edu for any questions

NPS@: Network Protein Sequence @nalysis

This site is a fork of the original PRABI NPS@ server

[HOME] [DESCRIPTION] [HELP] [NEWS] [CONTACT] [Geno3D]

July 30, 2024: **NPS@** updated (see NEWS).

In your publication cite :
NPS@: Network Protein Sequence Analysis
TIBS 2000 March Vol. 25, No 3 [291]:147-150
Combet C., Blanchet C., Geourjon C. and Deléage G.

Job **SOPMA** (ID: d275e591ad0d) submitted to **NPS@** server on 20250509-062735.

Queued.! Running.! Computed in 42 s !

SOPMA result for : liudafeng2017qqcom

Abstract Geourjon, C. & Deléage, G., SOPMA: Significant improvement in protein secondary structure prediction by consensus prediction from multiple alignments. Cabios, 1995, 11, 681-684.

```
       10        20        30        40        50        60        70
        |         |         |         |         |         |         |
MAMIITKQILRPSSPTPQAFKNHKLSYLDQIQAPIYIPLLFFYKNEESKYPDQISQRFKQSLSEILTIFY
eeeeeccceecccccccccchhhhhhhhhhcccccccceeeeeccccccchhhhhhhhhhhhhhhhhhhc
PLAGTMRHNSFVDCNDRGVEFVEVRVHARLAQFIQDPKMEELKQLIPVDCISHTDDDFLLLVKISYFDCG
ccccccccceeeecccccheeehhhhhhhhhhhhcccccchhhhhccccccccccccceeeeeeeeeccc
EVVVGVCMSHKIGDGISLAAFMNAWAATCRGESSSEIIHPSFDLALHFPPKDHLSSASSFRVAIAQENIM
ceeeeeehhhhhccchhhhhhhhhhhhhhccccccccccccccccccccccccccccccccccccccchh
TKRLVFDREKLEKLRKRIAASSDGVRDPSRVEAVSVFIWKSLIEAHKAESHMTETPAVSIASHAVNLRPR
hheeeechhhhhhhhhhhhhcccccccccchhhhhhhhhhhhhhhhhccccccccccccheeeehhhcccc
TVPQMDQTFGNCYAPASAVVSWDEDYVHHSRLRAALREIDDDYINKVLKADNNYLTQDQIGDLFKPENSV
ccccccccccceeehhhccccccccchhhhhhhhhhhhhhhhhhhhhhhccchhhhhhhhhhhcccccccce
LSSWWRFPVYKVDFGWGKPVWVSTTTIQYMNLIIFTSTPSEDGIEAWVTTTHNFFQVLQANYNKLDT
eecccccceeeecccccccceeeccccccccceeeeeecccccchee eeehhhhhhhhhhccchhhhh
```

Sequence length :   417

SOPMA :
- Alpha helix (Hh) : 155 is 37.17%
- 3_{10} helix (Gg) : 0 is 0.00%
- Pi helix (Ii) : 0 is 0.00%
- Beta bridge (Bb) : 0 is 0.00%
- Extended strand (Ee) : 66 is 15.83%
- Beta turn (Tt) : 0 is 0.00%
- Bend region (Ss) : 0 is 0.00%
- Random coil (Cc) : 196 is 47.00%
- Ambiguous states (?) : 0 is 0.00%
- Other states : 0 is 0.00%

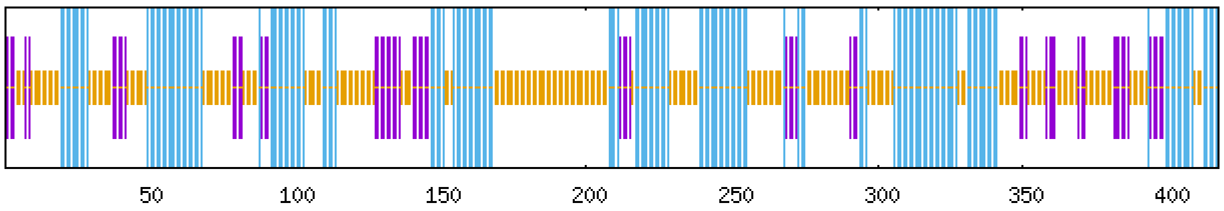

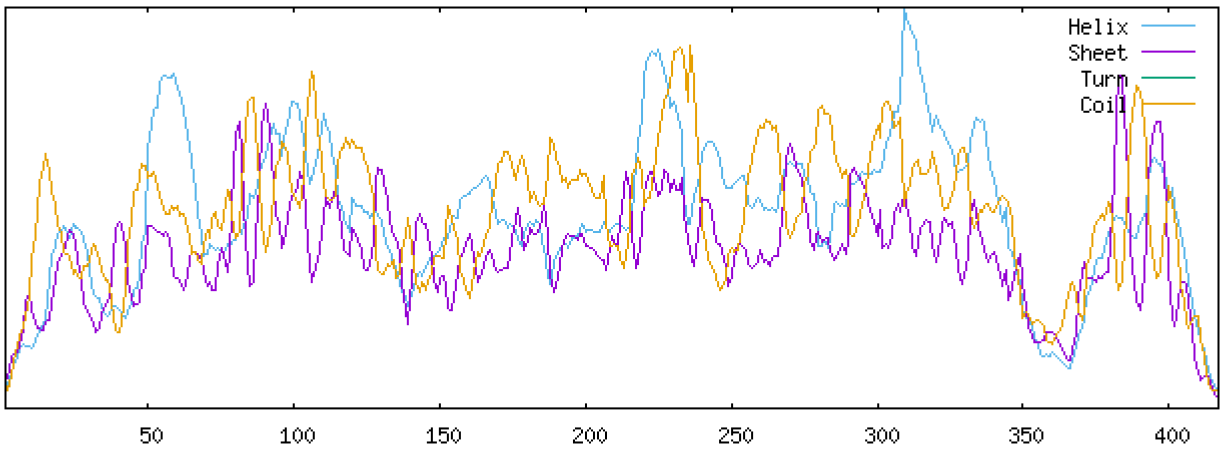

Parameters :
- Window width : 17
- Similarity threshold : 8
- Number of states : 3

Prediction result file (text): [SOPMA]
Intermediate result files (text): [PSI-BLAST on UniProtKB 50% identity] [KALIGN MSA in CLUSTAL W format]

***Last modification time :** Fri May 9 06:28:19 2025. **Current time :** Fri May 9 06:28:19 2025.* **User :** public@43.242.154.90.

© 1998-2025 Inserm Legal notice

This website uses cookies. By continuing to browse this site, you are agreeing to the use of our site cookies. To find out more, see our Terms of Use. OK

PDBsum entry gzs8

Chain A (417 residues)

UniProt code: A0A0K0LCG5 (A0A0K0LCG5_9LAMI) [Pfam]

Secondary structure:

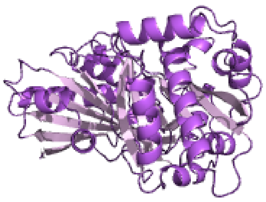

Protein chain A highlighted (click to view)

JSmol

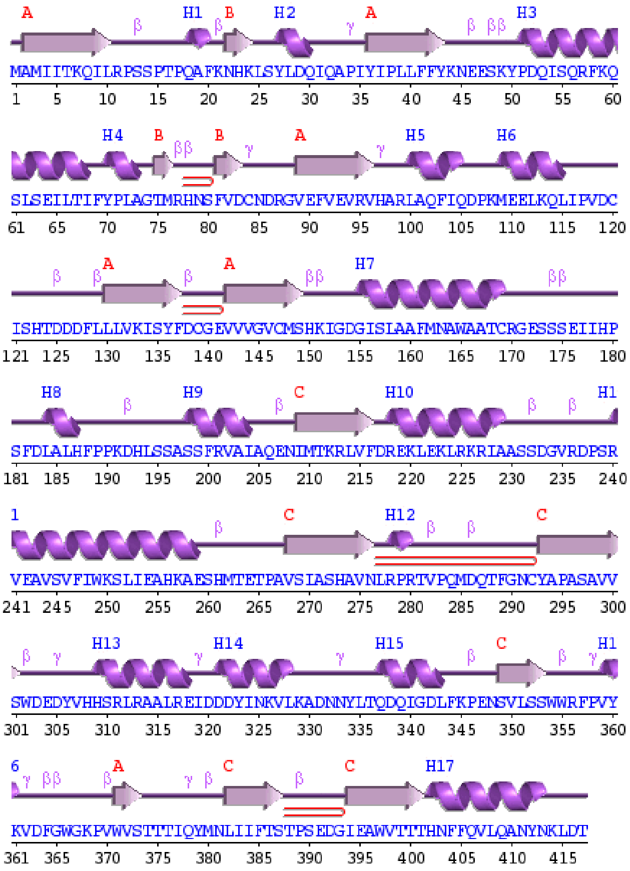

FASTA file

Topology

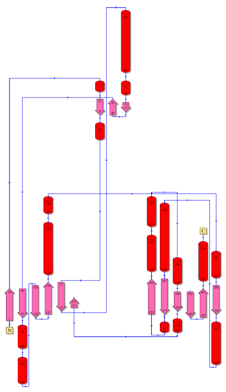

Hera

Motifs

Secondary structure

Wiring diagram

ProMotif

- 3 sheets
- 1 beta alpha beta unit
- 4 beta hairpins
- 1 psi loop
- 5 beta bulges
- 15 strands
- 17 helices
- 14 helix-helix interacs
- 29 beta turns
- 9 gamma turns

Key:

Sec. struc: Helix Strand — Helices labelled H1, H2, ... and strands by their sheets A, B, ...

Motifs: β beta turn γ gamma turn ⊂ beta hairpin

Related protein sequences in the PDB

EMBL-EBI, Wellcome Genome Campus, Hinxton, Cambridgeshire, CB10 1SD, UK +44 (0)1223 49 44 44

Copyright © EMBL-EBI 2025 | EBI is an outstation of the European Molecular Biology Laboratory | Terms of use

NPS@: Network Protein Sequence @nalysis

This site is a fork of the original PRABI NPS@ server

July 30, 2024: **NPS@** updated (see NEWS).

In your publication cite :
NPS@: Network Protein Sequence Analysis
TIBS 2000 March Vol. 25, No 3 [291]:147-150
Combet C., Blanchet C., Geourjon C. and Deléage G.

Job **SOPMA** (ID: 878ffb8c400a) submitted to **NPS@** server on 20250509-062829.

Queued! Running..! Computed in 40 s !

SOPMA result for : liudafeng2017qqcom

Abstract Geourjon, C. & Deléage, G., SOPMA: Significant improvement in protein secondary structure prediction by consensus prediction from multiple alignments. Cabios, 1995, 11, 681-684.

```
       10        20        30        40        50        60        70
        |         |         |         |         |         |         |
MASTKTLTFKVTRKNRELISPAEPTPYGFKYLSDIDDQDCLRFRFPLIFFYRENISMKGKDPIKIIRDAV
hccccche eeeeccccceeeccccccccchheehhhhhhccccccccceeeeecccccccccchhhhhhhhhh
```

MASTKTLTFKVTRKNRELISPAEPTPYGFKYLSDIDDQDCLRFRFPLIFFYRENISMKGKDPIKIIRDAV
hccccccheeeeeccccceeeccccccccchheehhhhhhccccccccceeeeeccccccccchhhhhhhhhh
AKALVFYYPFAGRLRECDSRKLVVDCTGEGVVFVEADADVMLQQFGDALHPPFPNLEKLLLDTPDYDGTI
hhhhhhhccccccccccccceeeeeccccceeeehhhhhhhhhhhccccccccchhhhhccccccccccc
NCPILFIQVTRLKCGGFILSYSCNHTICDAAGFVQFMSAVGELARGATAPSIQPVWERHLLTARNPPRVS
ccceeeeeeeeeccccceeeeeeechhhccchhhhhhhhhhhhhhcccccccccccccccceeccccccccc
FTHREYDVVPKTNGETDKMVVRYFFFDAADISALRRSLPRYLQTCSKFDIVAACAWRCRTIALSLKPDEE
cccccccccccccccccchhhheeeechhhhhhhhhhhcccccccccchhhhhhhhhhhhhhhhhcccccccc
VVFVNTVNIRNKMKPPLPVGYYGNGIVFPAVVTTAKKLSENPFQYAVELVMKGKYEATDDYVRSVADLMV
heeeeehcccccccccccccccccheeehhhhhccchhhccchhhhhhhhhhhhhhhhhhhhhhhhhhhhh
MRDRPSVGAGMNYYIVSDTSTAGFEEVEVGWGKPVYGGVAKGTIDWIGVNWYIPFKNKKGEQGKIVTVCL
ccchhhcccccceeeeecccccceeeeccccccceeeccccccccccceeeeeecccccccccceeeeeeh
PLNAMEEFAKQFRMMITAARTLNLSAL
hhhhhhhhhhhhhhhhhhhcccccccc

Sequence length : 447

SOPMA :
- Alpha helix (Hh) : 151 is 33.78%
- 3_{10} helix (Gg) : 0 is 0.00%
- Pi helix (Ii) : 0 is 0.00%
- Beta bridge (Bb) : 0 is 0.00%
- Extended strand (Ee) : 78 is 17.45%
- Beta turn (Tt) : 0 is 0.00%
- Bend region (Ss) : 0 is 0.00%
- Random coil (Cc) : 218 is 48.77%
- Ambiguous states (?) : 0 is 0.00%
- Other states : 0 is 0.00%

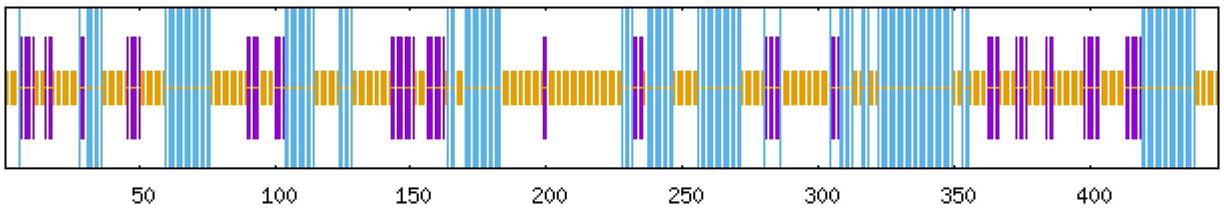

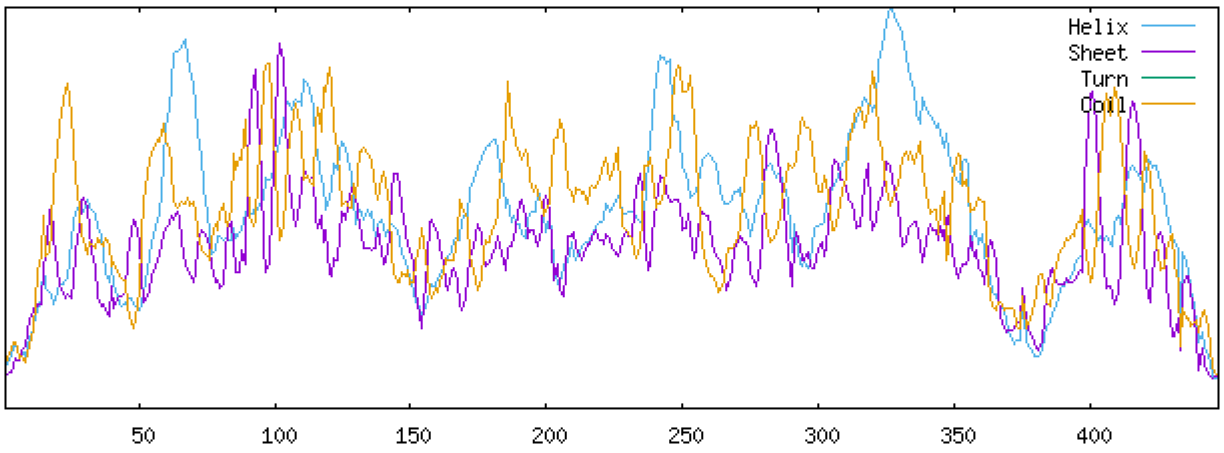

Parameters :
- Window width : 17
- Similarity threshold : 8
- Number of states : 3

Prediction result file (text): [SOPMA]
Intermediate result files (text): [PSI-BLAST on UniProtKB 50% identity] [KALIGN MSA in CLUSTAL W format]

***Last modification time :** Fri May 9 06:29:09 2025. **Current time :** Fri May 9 06:29:09 2025.* **User :** public@43.242.154.90.

© 1998-2025 Inserm UNIVERSITÉ DE LYON Legal notice

This website uses cookies. By continuing to browse this site, you are agreeing to the use of our site cookies. To find out more, see our Terms of Use. OK

PDBsum entry gzs9

Protein chain A

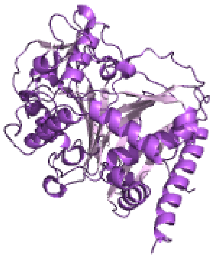

Protein chain A highlighted
(click to view)

JSmol

Motifs

Secondary structure

Wiring diagram

ProMotif

- 4 sheets
- 1 beta alpha beta unit
- 4 beta hairpins
- 1 psi loop
- 4 beta bulges
- 18 strands
- 18 helices
- 14 helix-helix interacs
- 31 beta turns
- 8 gamma turns

Chain A (447 residues)

UniProt code: A0A0K0LBP0 (A0A0K0LBP0_9LAMI) [Pfam]

Secondary structure:

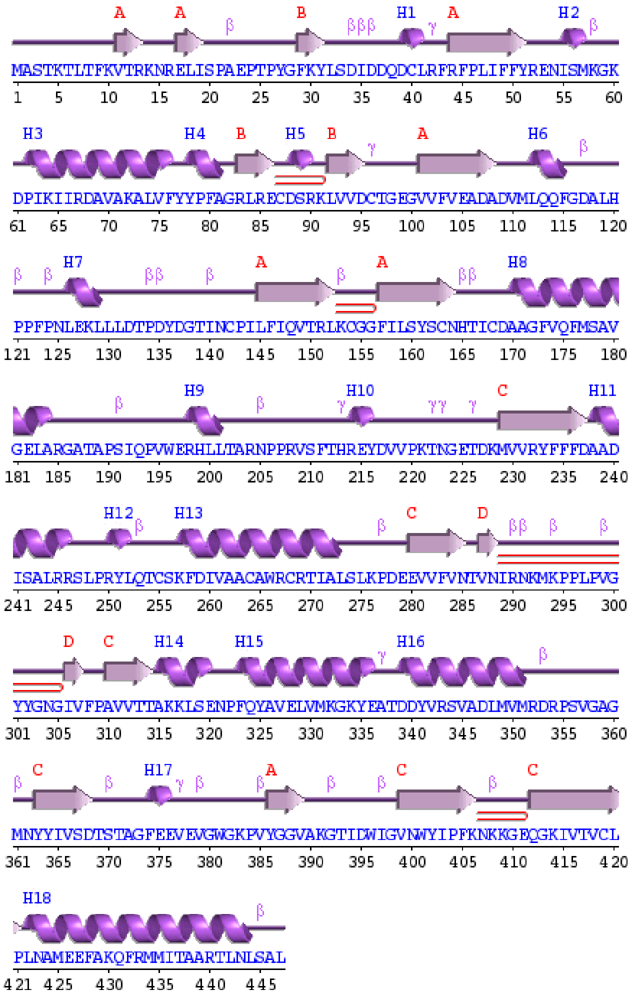

FASTA file

Topology

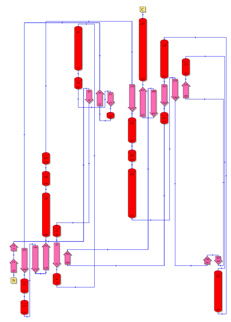

Hera

Key:

Sec. struc: Helix Strand — Helices labelled H1, H2, ... and strands by their sheets A, B, ...

Motifs: β beta turn γ gamma turn beta hairpin

Related protein sequences in the PDB

Visit us
Contact us

EMBL-EBI, Wellcome Genome Campus, Hinxton, Cambridgeshire, CB10 1SD, UK +44 (0)1223 49 44 44

Copyright © EMBL-EBI 2025 | EBI is an outstation of the European Molecular Biology Laboratory | Terms of use

```
                               1        10        20        30        40        50        60        70
tr|A0A0K0LBP0|A0A0K0LBP0_LAVIN .MASTKTLTFKVTRKNRELISPAEPTPYGFK.YLSDIDDQDCLRFRFPLIFFYRENISMK.GKDPIKIIRDAVAKAL
tr|D0QJ94|D0QJ94_VASCU         MAEKASSLMFNVRRHEPELITPAKPTPREIK.LLSDIDDQDGLRFQVPIIQFYKNNSSMQ.GKNPAKIIKSALAETL
sp|Q64FJ6|ATRGA_MALDO          ...MMSFSVLQVKRLQPELITPAKSTPQETK.FLSDIDDQESLRVQIPIIMCYKDNPSLNKNRNPVKAIREALSRAL
tr|Q6QLX4|Q6QLX4_SOLLC         ...MANILPISINYHKPKLVVPSSVTSHETK.RLSEIDDQGFIRLQIPILMFYKYNSSMK.GKDLAKIIKDGLSKTL
sp|P0DO25|AT9_ACTDE            ......MASSVRLVKKPVLVAPVDPTPSTVL.SLSSLDSQLFLRFPIEYLLVYASPHGVD.RAVTAARVKAALARSL
tr|A0A0K0LCG5|A0A0K0LCG5_LAVIN ..........MAMIITKQILRPSSPTPQAFKNHKLSYLDQIQAPIYIPLLFFYKNEESKY.PDQISQRFKQSLSEIL
tr|Q9FVF1|Q9FVF1_FRAAN         ......MEKIEVSINSKHTIKPSTSSTPLQP.YKLTLLDQLTPPAYVPIVFFYPITDHDFNLPQTLADLRQALSETL

                                 80        90       100       110       120       130       140       150
tr|A0A0K0LBP0|A0A0K0LBP0_LAVIN VFYYPFAGRLRECDSRK.LVVDCTGEGVVFVEADADVMLQQFGDALHPPFPNLEKLLLDTPDYDGTINCPILFIQVT
tr|D0QJ94|D0QJ94_VASCU         VHYYPLAGRLREGFGRK.LMVECTGEGILFIEADADVTLHEFGDDLPPPFPCLVELLYDVPGSSGIIDTPLLLIQVT
sp|Q64FJ6|ATRGA_MALDO          VYYYPLAGRLREGPNRK.LVVDCNGEGILFVEASADVTLEQLGDKILPPCPLLEEFLYNFPGSDGIIDCPLLLIQVT
tr|Q6QLX4|Q6QLX4_SOLLC         VFYYPLAGRLIEGPNKK.LMVNCNGEGVLFIEGDANIELEKLGESIKPPCPYLDLLLHNVHGSDGIIGSPLLLIQVT
sp|P0DO25|AT9_ACTDE            VPYYPLAGRVKTRPDSTGLDVVCQAQGAGLLEAVSDYTASDFQRAPRSVTEWRKLLLVEVFK.....VVPPLVVQLT
tr|A0A0K0LCG5|A0A0K0LCG5_LAVIN TIFYPLAGTMRHN.....SFVDCNDRGVEFVEVRVHARLAQFIQDPK...MEELKQLIPVDCISHTDDDFLLLVKIS
tr|Q9FVF1|Q9FVF1_FRAAN         TLYYPLSGRVKNN.....LYIDDFEEGVPYLEARVNCDMTDFLRLRKIECLNEFVPIKPFSMEAISDERYPLLGVQV

                                       160       170       180       190       200       210       220
tr|A0A0K0LBP0|A0A0K0LBP0_LAVIN RLKCGGFILSYSCNHTICDAAGFVQFMSAVGELARG...ATAPSIQPVWERHLLTARNPPRVSFTHREYDVVPKTNG
tr|D0QJ94|D0QJ94_VASCU         RLKCGGFIFALRLNHTMSDASGLVQFMTAVGEMARG...QRSLSIQPVWERHLLNARDPPRVTHIHHEYDDLEDTKG
sp|Q64FJ6|ATRGA_MALDO          CLTCGGFILALRLNHTMCDAAGLLLFLTAIAEMARG...AHAPSILPVWERELLFARDPPRITCAHHEYEDVIGHSD
tr|Q6QLX4|Q6QLX4_SOLLC         RFTCGGFAVGFRFNHTMMDAYGFKMFLNALSELIQG...ASTPSILPVWERHLLSARSSPSITCIHHEFDEEIESKI
sp|P0DO25|AT9_ACTDE            WLSDGCVALGVGFSHCVIDGIGSSEFLNLFAELATGRARLSEFQPKPVWDRHLLNSAG.RTNLGTHPEFGRVPDLSG
tr|A0A0K0LCG5|A0A0K0LCG5_LAVIN YFDCGEVVVGVCMSHKIGDGISLAAFMNAWAATCRGE..SSSEIIHPSFDLALHFPPKDHLSSASSFRVAIAQENIM
tr|Q9FVF1|Q9FVF1_FRAAN         NVFDSGIAIGVSVSHKLIDGGTADCFLKSWGAVFRG...CRENIIHPSLSEAALLFPPRDDLPEKYVDQMEALWFAG

                                          230       240       250       260       270       280       290
tr|A0A0K0LBP0|A0A0K0LBP0_LAVIN ........ETDKMVVRYFFFDAADISALRRSLP.RYLQTCSKFDIVAACAWRCRTIALSLKPDEEVVFVNTVNIRNK
tr|D0QJ94|D0QJ94_VASCU         T.....IIPLDDMVHRSFFFGPSEMAAIRRLVP.AHFHRSTTSEVLTAYLWRCYTIALQPDPEEEMRVICVVNSRTK
sp|Q64FJ6|ATRGA_MALDO          GSY..ASSNQSNMVQRSFYFGAKEMRVLRKQIPPHLISTCSTFDLITACLWKCRTLALNINPKEAVRVSCIVNARGK
tr|Q6QLX4|Q6QLX4_SOLLC         A....WESMEDKLIQQSFFFGNEEMEVIKNQVP..PNYECTKFELLMAFLWKCRTIALNLHSDEIVRLTYVINIRGK
sp|P0DO25|AT9_ACTDE            FVTRFTQERLSPTSITFDKTWLKELKNIAMSTSQPGEFPYTSFEVLSGHIWRSWARSLNLPAKQVLKLLFSINIRNR
tr|A0A0K0LCG5|A0A0K0LCG5_LAVIN T............KRLVFDREKLEKLRKRIAASSDGVRDPSRVEAVSVFIWKSLIEAHKAESHMTETPAVSIASHAV
tr|Q9FVF1|Q9FVF1_FRAAN         K..........KVATRRFVFGVKAISSIQDEAKSESVPKPSRVHAVTGFLWKHLIAASRALTSGTTSTRLSIAAQAV

                                     300       310       320       330       340       350
tr|A0A0K0LBP0|A0A0K0LBP0_LAVIN MK....PPLPVGYYGNGIVFPAVVTTAKKLSENPFQYAVELVMKGKYEATDDYVRSVADLMVMRDRPSV........
tr|D0QJ94|D0QJ94_VASCU         LN....PPLPTGFYGNGIAFPAAISQAKKICENPFGYTLQLVKQTKVDVTEEYMRSAADLMAMKGRPHF........
sp|Q64FJ6|ATRGA_MALDO          HNN...VRLPLGYYGNAFAFPAAISKAEPLCKNPLGYALELVKKAKATMNEEYLRSVADLLVLRGRPQY........
tr|Q6QLX4|Q6QLX4_SOLLC         KSLN..IELPIGYYGNAFITPVVVSKAGLLCSNPVTYAVELIKKVKDHINEEYIKSLIDLMVTKGRPEL........
sp|P0DO25|AT9_ACTDE            VKP....SLPAGYYGNAFVLGCAQTSVKDLTEKGLGYCADLVRGAKERVGDEYAREVVESVSWPRRASP........
tr|A0A0K0LCG5|A0A0K0LCG5_LAVIN NLRP..RTVPQMDQTFGNCYAPASAVVSWDEDYVHHSRLRAALREIDDDYINKVLKADNNYLTQDQIGD........
tr|Q9FVF1|Q9FVF1_FRAAN         NLRTRMNMETVLDNATGNLFWWAQAILELSHTTPEISDLKLCDLVNLLNGSVKQCNGDYFETFKGKEGYGRMCEYLD

                                       360       370       380       390       400       410       420
tr|A0A0K0LBP0|A0A0K0LBP0_LAVIN .......GAGMNYYIVSDTSTAGFEEVEVGWG..KPVYGGVAKGTIDWIG..VNWYIPFKNKKGEQGKIVTVCLPLN
tr|D0QJ94|D0QJ94_VASCU         .......TVVR.RYMVSDVTRAGFGLVDFGWGRPEPVYGGPAKGGVGPIPGVTSFFVPFKNRKGEKGIVVPTCLPTP
sp|Q64FJ6|ATRGA_MALDO          .......SSTGSYLIVSDNTRVGFGDVNFGWG..QPVFAGPVK.....ALDLISFYVQHKNN.TEDGILVPMCLPSS
tr|Q6QLX4|Q6QLX4_SOLLC         .......TKSWNFLVS.DNRYIGFDEFDFGWG..NPIFGGILK.....AISFTSFGVSVKNDKGEKGVLIAISLPPL
sp|P0DO25|AT9_ACTDE            .......DSVG.VLIISQWSRLGLDRVDFGLG..RPVQVGPICCDR........YCLFLPVRDRTESVKVMVAVPTS
tr|A0A0K0LCG5|A0A0K0LCG5_LAVIN .......LFKPENSVLSSWWRFPVYKVDFGWG..KPVWVSTTTIQY.......MNLIIFTSTPSEDGIEAWVTTTHN
tr|Q9FVF1|Q9FVF1_FRAAN         FQRTMSSMEPAPDIYLFSSWTNFFNPLDFGWG..RTSWIGVAGKIES....ASCKFIILVPTQCGSGIEAWVNLEEE

                                    430       440
tr|A0A0K0LBP0|A0A0K0LBP0_LAVIN AMEEFAKQFRMMITAARTLNLSAL.........
tr|D0QJ94|D0QJ94_VASCU         AMERFAKLMNEILQNQLLVSAEENKSVFIVSAI
sp|Q64FJ6|ATRGA_MALDO          AMERFQQELERITQEPKEDICNNLRSTSQ....
tr|Q6QLX4|Q6QLX4_SOLLC         AMKKLQDIYNMTFRVIISNI.............
sp|P0DO25|AT9_ACTDE            AVDRYEYFIRSPYS...................
tr|A0A0K0LCG5|A0A0K0LCG5_LAVIN FFQVLQANYNKLDT...................
tr|Q9FVF1|Q9FVF1_FRAAN         KMAMLEQDPHFLALASPKTLI............
```
